# Increased Multidrug-Resistant *Salmonella* *enterica* I Serotype 4,[5],12:i:- Infections Associated with Pork, United States, 2009–2018

**DOI:** 10.3201/eid2902.220950

**Published:** 2023-02

**Authors:** Ian D. Plumb, Allison C. Brown, Erin K. Stokes, Jessica C. Chen, Heather Carleton, Beth Tolar, Preethi Sundararaman, Amy Saupe, Daniel C. Payne, Hazel J. Shah, Jason P. Folster, Cindy R. Friedman

**Affiliations:** Centers for Disease Control and Prevention, Atlanta, Georgia, USA (I.D. Plumb, A.C. Brown, E.K. Stokes, J.C. Chen, H. Carleton, B. Tolar, P. Sundararaman, D.C. Payne, H.J. Shah, J.P. Folster, C.R. Friedman);; Minnesota Department of Health, St. Paul, Minnesota, USA (A. Saupe)

**Keywords:** Salmonella, bacteria, antimicrobial resistance, food safety, Salmonella 4,[5],12:i:-, Salmonella Typhimurium, pork, molecular epidemiology, United States

## Abstract

Reports of *Salmonella* *enterica* I serotype 4,[5],12:i:- infections resistant to ampicillin, streptomycin, sulphamethoxazole, and tetracycline (ASSuT) have been increasing. We analyzed data from 5 national surveillance systems to describe the epidemiology, resistance traits, and genetics of infections with this *Salmonella* strain in the United States. We found ASSuT-resistant *Salmonella* 4,[5],12:i:- increased from 1.1% of *Salmonella* infections during 2009–2013 to 2.6% during 2014–2018; the proportion of *Salmonella* 4,[5],12:i:- isolates without this resistance pattern declined from 3.1% to 2.4% during the same timeframe. Among isolates sequenced during 2015–2018, a total of 69% were in the same phylogenetic clade. Within that clade, 77% of isolates had genetic determinants of ASSuT resistance, and 16% had genetic determinants of decreased susceptibility to ciprofloxacin, ceftriaxone, or azithromycin. Among outbreaks related to the multidrug-resistant clade, 63% were associated with pork consumption or contact with swine. Preventing *Salmonella* 4,[5],12:i:- carriage in swine would likely avert human infections with this strain.

Nontyphoidal *Salmonella* infection is the leading cause of global foodborne illnesses (*1*,*2*) and is estimated to cause 1.35 million illnesses annually in the United States (*3*). *Salmonella enterica* I serotype 4,[5],12:i:- (hereafter 4,[5],12:i:-), is a monophasic variant of *Salmonella enterica* serotype Typhimurium lacking expression of the second phase flagellar antigen, which is encoded by the *fljB* gene (*4*,*5*). In the United States, 4,[5],12:i:- is the fifth most commonly reported *Salmonella* serotype causing human illness (*5*).

Infections caused by 4,[5],12:i:- have been increasingly reported since the 1990s (*4*,*6*). In recent years, a multidrug-resistant (MDR) strain of 4,[5],12:i:- has been described in Europe (*7*,*8*), Canada (*9*), Australia (*10*), and the United States (*11*). This MDR strain is characterized by resistance to ampicillin, streptomycin, sulphamethoxazole, and tetracycline (ASSuT). The ASSuT resistance pattern in this strain is encoded by a genomic region containing the antimicrobial drug resistance determinants *bla*_TEM-1B_, *strA-strB*, *sul2*, and *tet(B)*, in the usual location of an absent *fljB* gene (*11*,*12*). A separate genomic island (SGI-4) contains genes conferring tolerance to heavy metals (*8*,*11*).

Limited reports are available about the epidemiology of 4,[5],12:i:- in the United States. We used data from 5 national surveillance systems to describe the epidemiology, antimicrobial resistance, and molecular genetics of infections with 4,[5],12:i:- strains that were associated with ASSuT resistance.

## Methods

### Sources and Analysis of Surveillance Data

#### Laboratory-based Enteric Disease Surveillance System

The Laboratory-based Enteric Disease Surveillance (LEDS) system of the Centers for Disease Control and Prevention (CDC) collects information on *Salmonella* isolates collected from persons with infections and submitted to state and territorial public health laboratories in the United States (*5*). We summarized the incidence of 4,[5],12:i:- during 2009–2018 by using population estimates from the US Census (https://www.census.gov) as denominators and summarized the proportion of nontyphoidal *Salmonella* isolates that were 4,[5],12:i:-. Throughout this article, we use the term *Salmonella* to refer to nontyphoidal *Salmonella*; that is, excluding serotypes Typhi and Paratyphi A, C, and tartrate-negative B, which represent <2% of serotyped isolates (*5*).

#### PulseNet

PulseNet, the national molecular subtyping network for US foodborne disease surveillance, monitors strains of *Salmonella* and other enteric bacteria for potential outbreaks. During 1996–2019, participating laboratories performed molecular subtyping by pulsed-field gel electrophoresis (PFGE) on *Salmonella* isolates by using standard methods (*13*). Since 2015, these laboratories have increasingly performed whole-genome sequencing (WGS). After sequencing isolates using established protocols, participating laboratories analyze sequence data using BioNumerics Software (Applied Maths, http://www.applied-maths.com) (*14*,*15*). When sequences meet PulseNet quality metrics, participating laboratories upload sequence information to the CDC *Salmonella* PulseNet national database with other isolate data and upload a subset of this information to the National Center for Biotechnology Information (https://www.ncbi.nlm.nih.gov). We analyzed PulseNet data for sequenced 4,[5],12:i:- isolates collected during 2015–2018 that were concordant between genotypic serotyping by using SeqSero (http://www.denglab.info/SeqSero) and phenotypic serotyping. We used core genome multilocus sequence typing (cgMLST) to identify phylogenetic clustering of isolates within the PulseNet database. We then used the National Center for Biotechnology Information pathogen detection portal to determine the relationship of clustered isolates to sequences reported in other studies on the basis of single-nucleotide polymorphism analysis (*8*,*11*,*16*,*17*). For a subset of isolates, we determined whether SGI-4 or the *hin* or *iroB* genes were present, because these genetic markers can distinguish 4,[5],12:i:- from some previously reported strains (*11*). We identified antimicrobial resistance determinants (ARDs) and plasmid replicons among sequenced isolates by using previously described protocols (*18*) and assigned predicted antimicrobial resistance using ResFinder and PointFinder drug keys in the National Antimicrobial Resistance Monitoring System (NARMS; https://github.com/StaPH-B/resistanceDetectionCDC). For isolates collected during 2015–2018, we identified PFGE patterns that were specific for membership of a cgMLST clade associated with ASSuT resistance (the MDR clade). We then used these PFGE patterns as a marker of clade expansion during 2009–2018, including a period before WGS data were routinely available.

#### NARMS

NARMS is a collaboration between CDC, the US Food and Drug Administration (FDA), US Department of Agriculture (USDA), and state and local health departments that monitors antimicrobial resistance of enteric bacteria isolated from ill persons, retail meats, and food animals (*19*). As part of CDC NARMS, public health laboratories submit every 20th nontyphoidal *Salmonella* isolate. CDC performs antimicrobial susceptibility testing by broth microdilution for a standard panel of 14 antimicrobial drug agents and interprets results using Clinical and Laboratory Standards Institute criteria, when available. Testing for decreased susceptibility to azithromycin started in 2011 and is defined as an MIC of >0.12 μg/mL (*20*,*21*). We described antimicrobial resistance and changes in resistance patterns among 4,[5],12:i:- isolates collected as part of NARMS surveillance during 2009–2018.

#### Foodborne Diseases Active Surveillance Network

The Foodborne Diseases Active Surveillance Network (FoodNet) conducts population-based active surveillance for laboratory-confirmed enteric infections, including *Salmonella* (*22*). FoodNet is a collaboration of CDC, 10 state health departments, USDA Food Safety and Inspection Service, and FDA. For each reported *Salmonella* infection, FoodNet collects data on patient demographics, hospitalizations, international travel in the 7 days before illness onset, and whether the infection was associated with a recognized outbreak. Since 2014, FoodNet has also collected data on consumption of pork, chicken, turkey, and beef during the 7 days before illness onset. We compared characteristics of 4,[5],12:i:- infections to other *Salmonella* serotype infections during 2009–2018. After linking FoodNet data with PulseNet data, we compared characteristics of 4,[5],12:i:- infections with PFGE patterns in the MDR clade to 4,[5],12:i:- infections with other PFGE patterns.

#### National Outbreak Reporting System

The National Outbreak Reporting System (NORS) collects information about outbreaks transmitted by food, water, environmental sources, infected persons or animals, or unknown modes of transmission (*23*). State, local, and territorial public health agencies submit reports to CDC that document outbreak characteristics, food and animal sources, and the pathogens that caused each outbreak. We analyzed NORS data collected during 2009–2018, and obtained PFGE data by linking with PulseNet. We compared the characteristics of outbreaks from 4,[5],12:i:- with outbreaks from other *Salmonella* serotypes. Among 4,[5],12:i:- outbreaks, we compared outbreaks by whether the PFGE pattern was found in the MDR clade identified in PulseNet.

### Statistical Methods

We used a 2-sample test of proportions to compare characteristics of 4,[5],12:i:- infections between 2009–2013 and 2014–2018. We used logistic regression to compare characteristics of *Salmonella* infections and outbreaks by 4,[5],12:i:- serotype and association with the cgMLST clade; we adjusted multivariable logistic regression models for age, sex, race, and ethnicity. We conducted statistical analyses by using SAS version 9.3 (SAS Institute Inc., https://www.sas.com) and Stata 15.0 (StataCorp LLC, https://www.stata.com).

## Results

### Incidence of Reported Infections

Among *Salmonella* isolates reported to LEDS during 2009–2018, a total of 19,212 (4.3%) were 4,[5],12:i:-, and 4,[5],12:i:- was reported from all 50 states. This total represented an average annualized incidence of 0.60 isolates per 100,000 persons. From 2009–2013 to 2014–2018, the frequency of 4,[5],12:i:- increased from 3.7% (7,828 isolates) to 4.9% (11,384 isolates) of reported *Salmonella* (p<0.001); thus, the annualized incidence increased from 0.50 to 0.71 reported infections per 100,000 persons (Appendix 1 Figure 1).

### Genotype Characteristics

For the 2015–2018 timeframe, we included 3,036 sequenced 4,[5],12:i:- isolates from the PulseNet database in the analysis (Appendix 2). Of those, 73% (2,215 isolates) had genetic resistance determinants, and 13% (404 isolates) had predicted resistance to antimicrobial drugs recommended as empiric treatment (first-line agents) for invasive *Salmonella* infections: 11% (320 isolates) were resistant to ciprofloxacin, 4.4% (135 isolates) to ceftriaxone, and 0.5% (14 isolates) to azithromycin (Appendix 1 Table 1). Ciprofloxacin resistance was conferred by the *qnr*B1 gene in 69% (222/320) of isolates; ceftriaxone resistance was conferred most frequently by *bla*_CMY-2_ (44%; 60/135 isolates) or *bla*_SHV-12_ (34%; 46/135 isolates). Decreased susceptibility to azithromycin was conferred most frequently by *mph*(*A*) (64%; 9/14 isolates). Genes conferring ASSuT resistance were detected in 54% (1,645) of isolates, of which 96% (1,579) carried *aph(3′′)-Ib*, *aph(3′)-Ia*, *aph(*6*)-Id*, *bla*_TEM-1B_, *sul2*, and *tet*(*B*). Five isolates had determinants of resistance to colistin: 3 isolates had *mcr*-1, 1 had *mcr*-3, and 1 had *pmrB* (Appendix 1 Table 1).

Overall, 69% (2,087/3,036) of 4,[5],12:i:- isolates were part of the MDR clade (median 18 alleles between isolates, range 0–108) (Appendix 1 Figure 2). Within this clade, 77% (1,612/2,087) of isolates had genetic determinants of ASSuT resistance, and 75% (1,561/2,087) had the same combination of *aph(3′′)-Ib*, *aph(3′)-Ia*, *aph(*6*)-Id*, *bla*_TEM-1B_, *sul2*, and *tet*(*B*); this set of genes was found in only 1.9% (18/949) of isolates outside the MDR clade. Among 4,[5],12:i:- isolates in the clade, 16% (333/2,087) had resistance determinants against first-line antimicrobial agents (ciprofloxacin, ceftriaxone, or azithromycin), compared with 7.5% (71/945) outside the clade (p<0.001). Isolates of 4,[5],12:i:- in the MDR clade were more likely to contain determinants conferring resistance to ciprofloxacin (13% vs. 5.2%; p<0.001) or ceftriaxone (5.4% vs. 2.3%; p<0.001), compared with isolates outside the clade (Appendix 1 Table 2).

Among isolates in the MDR clade, 2,008 (96%) belonged to the same single-nucleotide polymorphism cluster (PDS000076482.92) as previously reported ASSuT-resistant strains (*8*,*11*,*16*). Among a subset of 102 (5.1%) 4,[5],12:i:- isolates in the MDR clade, SGI-4 was detected in all isolates, *iroB* was detected in 99%, and *hin* was absent in 99%.

For isolates with both PFGE and WGS information, 275 PFGE patterns were identified in isolates within the MDR clade. MDR-associated PFGE patterns were detected in only 0.8% (7/937) of isolates outside the MDR clade. During 2009–2018, the proportion of 4,[5],12:i isolates with MDR-associated PFGE patterns increased from 29% (2,424/8,455) during 2009–2013 to 60% (6,900/11,546) during 2014–2018 (p<0.001) (Figure).

**Figure:**
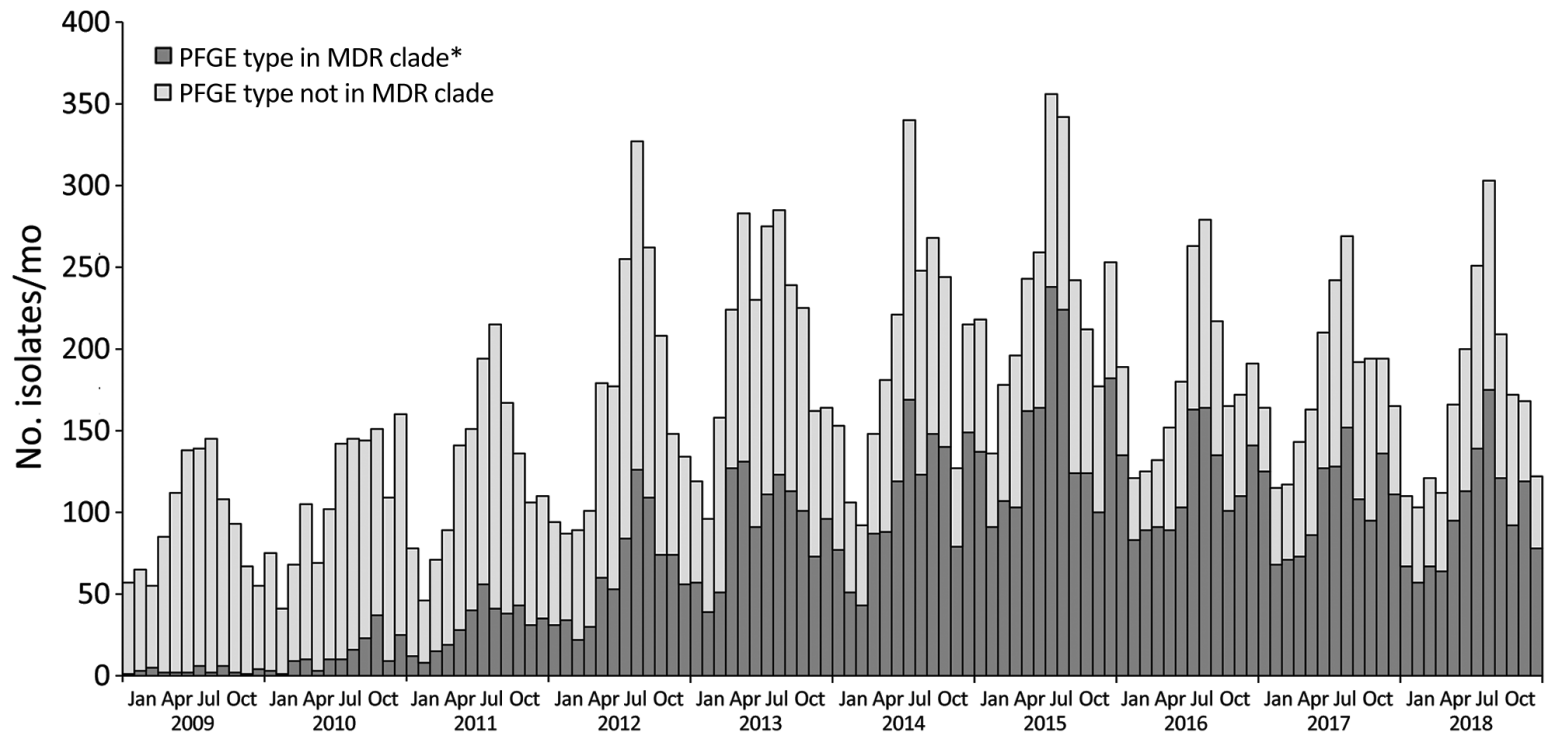
Clinical multidrug-resistant *Salmonella enterica* I serotype 4,[5],12:i:- in a study of infections associated with pork, United States, 2009–2018. Isolates were identified by whether PFGE pattern was linked to the multidrug resistant cgMLST clade. Among 3,007 sequenced clinical PulseNet isolates during 2015–2018 that had PFGE information, 69% (n = 2,070) belonged to a cgMLST clade associated with multidrug resistance containing 275 PFGE patterns; those patterns also were noted in 0.8% (7/937) of isolates outside the clade. cgMLST, core genome multilocus sequence type; PFGE, pulse-field gel electrophoresis.

### Antimicrobial Susceptibility Testing

During 2009–2018, 4.7% (1,079/23,175) of *Salmonella* isolates included in NARMS surveillance were 4,[5],12:i:-, of which 57% (613 isolates) had decreased susceptibility to antimicrobial drugs, 41% (440 isolates) had a resistance pattern that included ASSuT, and 35% (373 isolates) had only ASSuT resistance. From 2009–2013 to 2014–2018, the percentage of *Salmonella* isolates that were 4,[5],12:i:- increased from 4.3% (476/11,139) to 5.0% (603/12,036; p = 0.009), and the percentage that were 4,[5],12:i:- with ASSuT resistance increased from 1.1% (126/11,139) to 2.6% (p<0.001); meanwhile, the proportion that were 4,[5],12:i:- without ASSuT resistance declined from 3.1% (350/11,139) to 2.4% (289/12,036; p<0.001). Similarly, the percentage of *Salmonella* isolates that were 4,[5],12:i:- with PFGE patterns in the MDR clade increased, whereas the percentage that were 4,[5],12:i:- without those PFGE patterns declined (Appendix 1 Table 3, Figures 3, 4).

Overall, 8.4% (91/1,079) of 4,[5],12:i:- isolates had decreased susceptibility to first-line antimicrobial drugs: ciprofloxacin (6.1%, 66 isolates), ceftriaxone (2.9%, 31 isolates), or azithromycin (0.3%, 3 isolates). The percentage of 4,[5],12:i:- isolates that had decreased susceptibility to these drugs increased from 3.4% (16/476) during 2009–2013 to 12% (75/603) during 2014–2018 (p<0.001), including an increase in ciprofloxacin resistance from 1.1% (5 isolates) to 10% (66 isolates; p<0.001). Ciprofloxacin resistance increased between these periods, both among 4,[5],12:i:- isolates with PFGE patterns found in the MDR clade (1.0% to 7.4%; p<0.001), and among 4,[5],12:i:- isolates without MDR-associated PFGE patterns (1.8% to 12%; p = 0.001) (Appendix 1 Tables 3, 4).

### Case Characteristics

During 2009–2018, FoodNet sites reported 3,772 infections caused by 4,[5],12:i:-. Among infections with specified patient information, 29% (1,053/3,666) were in patients admitted to a hospital overnight, 4.1% (154/3772) of patients had bacteremia, and 0.5% (17/3,669) of patients died. Compared with other *Salmonella* infections, 4,[5],12:i:- infection was less frequently associated with international travel (adjusted odds ratio [aOR] 0.72, CI 0.62–0.83) (Table 1). Infection with 4,[5],12:i:- was more likely in the 1–18 year age group (aOR 1.83, 95% CI 1.58–2.12; referent age group <1 year of age), and for persons whose race and ethnicity was Black, non-Hispanic (aOR 1.29, 95% CI 1.17–1.42), other non-White/non-Hispanic (aOR 1.44, 95% CI 1.27–1.63), or Hispanic (aOR 1.28, 95% CI 1.15–1.42) (Table 1).

**Table 1:** Characteristics of multidrug-resistant *Salmonella enterica* 4,[5],12:i:- and other nontyphoidal *Salmonella* infections, United States, 2009–2018*

Characteristic	*Salmonella* 4,[5],12:i:-, n = 3,772	Other nontyphoidal *Salmonella*, n = 65,518	Odds ratio (95% CI)	Adjusted odds ratio (95% CI)†
Age group, y	N = 3,772	N = 65,509		
<1	258 (6.8)	6,157 (9.4)	Referent	Referent
1–17	1,440 (38.2)	18,614 (28.4)	1.85 (1.61–2.11)	1.83 (1.58–2.12)
18–64	1,618 (42.9)	31,493 (48.1)	1.23 (1.07–1.40)	1.25 (1.08–1.45)
>65	456 (12.1)	9,245 (14.1)	1.18 (1.01–1.38)	1.27 (1.07–1.50)
Sex	N = 3,771	N = 65,424		
M	1,887 (50)	30,825 (47.1)	Referent	Referent
F	1,884 (50)	34,599 (52.9)	0.89 (0.83–0.95)	0.93 (0.86–0.99)
Race and ethnicity	N = 3,728	N = 54,505		
White, non-Hispanic	1,916 (58.5)	36,240 (66.5)	Referent	Referent
Black, non-Hispanic	559 (17.1)	7,769 (14.3)	1.36 (1.23–1.50)	1.29 (1.17–1.42)
Hispanic	485 (14.8)	6,634 (12.2)	1.38 (1.25–1.53)	1.28 (1.15–1.42)
Other, non-Hispanic	318 (9.7)	3,862 (7.1)	1.56 (1.38–1.76)	1.44 (1.27–1.63)
International travel	220 (6.7), n = 3,275	4,848 (9.4), n = 51,516	0.69 (0.6–0.8)	0.72 (0.62–0.83)
Clinical outcome	N = 3,669	N = 63,696		
Hospitalized	1,053 (28.7)	18,219 (28.7)	1.00 (0.93–1.08)	1.03 (0.95–1.12)
Died	17 (0.5)	260 (0.4)	1.14 (0.69–1.86)	1.15 (0.67–1.99)

*Values are no. (%) except as indicated. †Adjusted for age, sex, race, and ethnicity.

Of 3,160 (84%) 4,[5],12:i:- infections with PFGE information, 1,427 (45%) had PFGE patterns found in the MDR clade. Among 4,[5],12:i:- infections, those linked to the MDR clade were associated with the 18–64 years of age group (aOR 1.42, 95% CI 1.02–1.97). Infections linked to the MDR clade also were associated with being Black and non-Hispanic (aOR 1.66, 95% CI 1.34–2.06), other non-White race with non-Hispanic ethnicity (aOR 2.46, 95% CI 1.88–3.23), having Hispanic ethnicity (aOR 2.26, 95% CI 1.80–2.83), and international travel (aOR 1.62, CI 1.17–2.24) (Table 2).

**Table 2:** Characteristics of multidrug-resistant *Salmonella enterica* 4,[5],12:i:- infections, United States, 2009–2018*

Characteristics	PFGE link to MDR clade		Odds ratio (95% CI)	Adjusted odds ratio (95% CI)†
	Linked, n = 1,427	Not linked, n = 1,733		
Age group, y				
<1	90 (6.3)	119 (6.9)	Referent	Referent
1–17	529 (37.1)	661 (38.1)	1.06 (0.79–1.42)	1.06 (0.76–1.47)
18–64	641 (44.9)	738 (42.6)	1.15 (0.86–1.54)	1.42 (1.02–1.97)
>65	167 (11.7)	215 (12.4)	1.03 (0.73–1.44)	1.28 (0.88–1.86)
Sex				
M	704 (49.3)	874 (50.4)	Referent	Referent
F	723 (50.7)	859 (49.6)	1.04 (0.91–1.20)	1.02 (0.88–1.19)
Race and ethnicity	N = 1,259	N = 1,494		
White, non-Hispanic	643 (51.1)	995 (66.6)	Referent	Referent
Black, non-Hispanic	225 (17.9)	219 (14.7)	1.59 (1.29–1.96)	1.66 (1.34–2.06)
Hispanic	235 (18.7)	174 (11.6)	2.09 (1.68–2.60)	2.26 (1.8–2.83)
Other, non-Hispanic	156 (12.4)	106 (7.1)	2.28 (1.75–2.97)	2.46 (1.88–3.23)
International travel	107 (8.4), n = 1,277	78 (5.2), n = 1,499	1.67 (1.23–2.25)	1.62 (1.17–2.24)
Clinical outcome				
Hospitalized	389 (27.8), n = 1,401	488 (29), n = 1,680	0.94 (0.80–1.10)	0.87 (0.73–1.04)
Died	7 (0.5), n = 1,392	7 (0.4), n = 1,685	1.21 (0.42–3.46)	1.40 (0.43–4.54)

*Values are no. (%) except as indicated. Values exclude 612 isolates without PFGE information. Among sequenced clinical PulseNet isolates collected during 2015–2018 that also had PFGE information, 2,070/3,007 (69%) belonged to a clade associated with ASSuT resistance that contained 275 PFGE patterns; these patterns were identified in 7/937 (0.8%) isolates outside the clade. ASSuT, ampicillin, streptomycin, sulphamethoxazole, and tetracycline; MDR, multidrug resistant; PFGE, pulsed-field gel electrophoresis. †Adjusted for age, sex, race, and ethnicity.

Pork consumption was more frequently associated with serotype 4,[5],12:i:- compared with other *Salmonella* infections (aOR 1.39, CI 1.18–1.65), and among 4,[5],12:i:- infections, consumption of pork was associated with PFGE patterns found in the MDR clade (aOR 1.86, CI 1.29–2.67). Those associations persisted after we excluded the 222 (5.9%) cases linked to recognized outbreaks (Appendix 1 Table 5).

### Outbreaks

During 2009–2018, 123 outbreaks caused by 4,[5],12:i:- were reported from 37 states, comprising 2,004 reported outbreak-associated illnesses, 295 hospitalizations, and 3 deaths. The percentage of *Salmonella* outbreaks attributed to 4,[5],12:i:- increased from 4.5% (42/926) during 2009–2013 to 7.9% (81/1,026) during 2014–2018 (OR 1.80, CI 1.23–2.65) (Appendix 1 Figure 5). Among the 123 outbreaks of 4,[5],12:i:- infections, 61% (75 outbreaks) were linked to food, 15% (18 outbreaks) to animal contact, and 5.7% (7 outbreaks) to transmission from other humans; 19% (23 outbreaks) had an unknown transmission mode (Table 3).

**Table 3:** Characteristics of outbreaks caused by multidrug-resistant *Salmonella enterica* 4,[5],12:i:- compared with other nontyphoidal *Salmonella*, United States, 2009–2018*

Outbreak type and transmission mode	*Salmonella* 4,[5],12:i:-, n = 123	Other *Salmonella*, n = 1,829	PFGE link to MDR clade	
			Linked, n = 4	Not linked, n = 45
Multistate outbreak	13 (10.6)	284 (15.5)	3 (4.1)	10 (22.2)
Mode of transmission				
Foodborne, no single food category†	52 (42.3)	792 (43.3)	25 (33.8)	26 (57.8)
Foodborne, single food category	23 (18.7)	414 (22.6)	17 (23)	5 (11.1)
Animal contact, no single animal type	6 (4.9)	15 (0.8)	4 (5.4)	2 (4.4)
Animal contact, single animal type	12 (9.8)	138 (7.5)	8 (10.8)	4 (8.9)
Person-to-person	7 (5.7)	126 (6.9)	6 (8.1)	1 (2.2)
Environmental	0	15 (0.8)	0	0
Waterborne	0	2 (0.1)	0	0
Unknown transmission	23 (18.7)	327 (17.9)	14 (18.9)	7 (15.6)
Food source, single food category	N = 23	N = 406	N = 17	N = 5
Chicken	4 (17.4)	77 (19)	3 (17.6)	1 (20)
Pork	13 (56.5)	47 (11.6)	11 (64.7)	1 (20)
Turkey	2 (8.7)	27 (6.7)	2 (11.8)	0
Other food category	4 (17.4)‡	255 (62.8), n = 406	1 (5.9), n = 17	3 (60)
Animal contact, single animal type	N = 12	N = 138	N = 8	N = 4
Live poultry	3 (25.0)§	77 (55.8)	2 (25.0)	1 (25)
Swine	5 (41.7)	0	5 (62.5)	0
Other animal	4 (33.3)¶	61 (44.2)	1 (12.5)	3 (75)

*Values are no. (%) except as indicated. PFGE used as a marker for the MDR clade as defined by core genome multilocus sequence typing in PulseNet. Among sequenced clinical PulseNet isolates collected during 2015–2018 that also had PFGE information, 2,070/3,007 (69%) belonged to a clade associated with ASSuT resistance that contained 275 PFGE patterns; these patterns were identified in 7/937 (0.8%) isolates outside the clade. Excludes 4 outbreaks without PFGE information. ASSuT, ampicillin, streptomycin, sulphamethoxazole, and tetracycline; MDR, multidrug resistant; PFGE, pulsed-field gel electrophoresis. †Includes confirmed 4,[5],12:i:- outbreaks classified as foodborne transmission for which no specific food was reported. ‡Includes dairy (n = 1), nuts or seeds (n = 1), seeded vegetables (n = 1), and sprouts (n = 1). §Linked to contact with chicken (n = 1), turkey (n = 1), or baby chick or duckling (n = 1). ¶Linked to contact with rodents (n = 2), turtles (n = 1), or cattle (n = 1).

Of 114 4,[5],12:i:- outbreaks with PFGE information, 62.2% (74 outbreaks) had PFGE patterns found in the MDR clade. Among these 114 outbreaks, 22 were linked to a single food source, of which 12 were linked to pork; among those 12, 11 had PFGE patterns found in the MDR clade. Among 12 outbreaks linked to contact with a single animal, 5 were linked to contact with swine; all 5 had PFGE patterns found in the MDR clade. Foodborne outbreaks of 4,[5],12:i:- infections with PFGE patterns in the MDR clade were also linked to consumption of chicken, turkey, and a dairy product. Additional outbreaks were linked to contact with live poultry and cattle. Compared with 4,[5],12:i:- outbreaks showing other PFGE patterns, 4,[5],12:i:- outbreaks with PFGE patterns found in the MDR clade were more likely to be linked to consumption of pork or contact with swine (64% [16/25] vs. 11% [1/9]; p = 0.007) (Table 3).

## Discussion

In the United States, nearly 20,000 reported infections were caused by 4,[5],12:i:- during 2009–2018, and >100 outbreaks were reported across 37 states. An overall increase in 4,[5],12:i:- infections was driven by expansion of a phylogenetic clade associated with ASSuT resistance (the MDR clade). By 2015–2018, a total of 69% of 4,[5],12:i:- isolates belonged to the MDR clade. That clade also contained a substantial proportion of isolates that might not respond to antimicrobial drugs recommended as empiric treatment for invasive *Salmonella* infections (*24*,*25*).

Isolates in the MDR clade in this study were closely related to isolates previously reported in Europe (*7*,*8*,*12*,*26*), the United States (*11*), Canada (*9*), Australia (*10*), and Japan (*27*) and had the same genes conferring ASSuT resistance (*11*,*12*,*16*) and the same genomic island conferring tolerance to heavy metals (*8*,*11*,*16*). Using PFGE data as a proxy for clade membership before the introduction of routine WGS, we found evidence that infections caused by this clade have been increasing in the United States since 2010. This finding is consistent with recent phylogenetic evidence that the strain was likely transmitted from Europe to the United States multiple times during 2000–2006 (*11*,*28*).

Infections in the MDR clade were more likely than other 4,[5],12:i:- isolates to have genetic determinants of resistance to ciprofloxacin or ceftriaxone. During 2015–2018, a total of 16% of sequenced isolates had predicted decreased susceptibility to ciprofloxacin, ceftriaxone, or azithromycin; beyond those antimicrobial drugs, few alternative agents are available to treat invasive *Salmonella* infections (*24*,*25*). Moreover, some isolates in the clade contained extended-spectrum β-lactamase genes conferring resistance to ceftriaxone or *mcr* genes conferring colistin resistance, consistent with reports from other countries (*10*,*11*,*29*,*30*).

Infections related to the MDR clade were more likely to occur among persons from ethnic and racial minority groups. The reasons for this association are unknown but might reflect differences in consumption of contaminated food, food preparation, or animal contact and warrants further investigation. The low proportion of infected persons with a history of international travel suggests that the main source of infections from the MDR clade was within the United States.

Our analysis indicates that infections from the MDR clade resulted from consumption of contaminated pork or contact with swine. Pork was the food most often implicated in 4,[5],12:i:- outbreaks and was more likely to be implicated compared with other *Salmonella* outbreaks. Among outbreaks of 4,[5],12:i:- infections linked to pork consumption or contact with swine, 89% were caused by strains with PFGE types that were characteristic of the MDR clade. Among outbreaks caused by these strains, 62% of those linked to a single food or animal were linked to pork or contact with swine; other outbreaks were linked to contact with poultry or cattle. In FoodNet sites, 4,[5],12:i:- infections with PFGE patterns that were characteristic of the MDR clade were more likely to be reported in patients who ate pork within 7 days before their illness onset than were other 4,[5],12:i:- or *Salmonella* infections. Collectively, our findings are consistent with previous reports indicating that swine is the predominant source of ASSuT-resistant 4,[5],12:i:- infections (*8*,*11*,*26*,*31*,*32*). Those studies also identified poultry and cattle as sources of ASSuT-resistant 4,[5],12:i:-, suggesting movement of this clade between animal reservoirs (*8*,*11*,*26*,*33*).

ASSuT-resistant strains can persist in swine herds (*34*), survive in swine feces (*35*), contaminate animal environments, including animal feed (*36*), and form biofilms (*36*). The 4,[5],12:i:- MDR clade might have a selective advantage in swine because of use of antimicrobial drugs or heavy metals. Use of tetracycline in swine production can select for tetracycline-resistant enteric bacteria (*37*), and ≈2 million cubic kilograms of tetracycline were sold for use in swine in the United States in 2019 (*38*). In this study, ciprofloxacin resistance was mainly encoded by *qnr*B19, a plasmid-mediated gene that confers resistance to enrofloxacin (*39*), a quinolone approved for use in US swine since 2008 (*40*). Resistance to enrofloxacin increased among *Salmonella* in swine during 2006–2015 (*41*), and similar ASSuT-resistant 4,[5],12:i:- isolates in swine have been reported that also contain *qnr*B19 (*42*). This gene might be acquired from other bacteria during swine production cycles (*39*). Another defining characteristic of this strain is the presence of SGI-4, which confers heavy metal tolerance (*8*,*11*,*43*). The reported frequent use of metals, such as copper and zinc, for disease prevention and growth promotion, might also provide a selective advantage (*40*,*44*,*45*).

One limitation of our study is that some of the reported increase in 4,[5],12:i:- infections might have resulted from improved serotyping, which can be difficult to perform (*46*). To address this concern, we limited our analysis to a recent period when serotyping was more reliable. In addition, misclassifying 4,[5],12:i:- as Typhimurium, or vice versa, would not explain the selective increase in isolates related to the MDR clade. Another limitation is that we were not able to verify the presence of the clade directly in several surveillance systems. Instead, we relied on PFGE patterns as a proxy for likely membership of the MDR clade in the outbreak and FoodNet surveillance systems and for isolates in the PulseNet database collected before 2015. We were not able to explain associations of the MDR clade with race, ethnicity, or other characteristics. The analyses depended implicitly on submission of isolates and data to national surveillance systems.

Our findings indicate that 4,[5],12:i:- infections have increased in the United States because of an MDR clade that has expanded since 2010. Illness is likely to have resulted from transmission from swine that carry it. The potential health threat from carriage among swine is highlighted by the recent identification of a 4,[5],12:i:- isolate from a retail pork specimen that contained extensive antimicrobial resistance genes (*47*). Further selection for this strain in swine might be prevented by limiting unnecessary agricultural use of classes of antibiotics to which the strain has resistance and by limiting unnecessary use of heavy metals in feed. Human illness from 4,[5],12:i:- also might be prevented by strategies to reduce carriage of 4,[5],12:i:- in swine, such as through the development and use of vaccines targeting 4,[5],12:i:- (*39*,*40*), and by improved husbandry and biosecurity measures (*48*). After an outbreak of the MDR clade that was attributed to contaminated whole hogs, a second outbreak occurred despite changes in practices at the slaughter facility, and inability to conduct on-farm investigations was cited as a major limitation (*49*). Prevention of human illness from this strain is likely to depend on addressing gaps in understanding at the preharvest stage.

A substantial proportion of 4,[5],12:i:- isolates have resistance to antimicrobial agents recommended as empiric treatment for invasive *Salmonella* infections. Consumers can minimize the risk for infection from contaminated products by safe food preparation and by avoiding consumption of undercooked pork. Clinicians caring for patients with suspected invasive salmonellosis or risk factors for invasive disease can confirm the diagnosis using stool culture and request antimicrobial susceptibility testing to guide therapy. Public health professionals can enact measures to identify clinically relevant resistance determinants that might emerge within this growing subgroup of 4,[5],12:i:- infections (*50*).

Appendix 1 Additional information increased multidrug-resistant *Salmonella enterica* I serotype 4,[5],12:i:- infections associated with pork, United States, 2009–2018.

Appendix 2 Whole-genome sequence serotype, multidrug resistance clade, and ASSuT pattern of *Salmonella enterica* I serotype 4,[5],12:1:- isolates from infections associated with pork, United States, 2009–2018.
