# Supplementary material for: Increased Multidrug-Resistant Salmonella enterica I Serotype 4,[5],12:i:- Infections Associated with Pork, United States, 2009–2018
Source: Emerg Infect Dis. 2023 Feb;29(2):314–22. doi: 10.3201/eid2902.220950 (PMC9881761; doi:10.3201/eid2902.220950)
Supplement: Appendix 1 — Additional information increased multidrug-resistant Salmonella enterica I serotype 4,[5],12:i:- infections associated with pork, United States, 2009–2018. [file 22-0950-Techapp-s1.pdf]

Article DOI: https://doi.org/10.3201/eid2902.220950

Increased Multidrug-Resistant *Salmonella enterica* I Serotype 4,[5],12:i:- Infections Associated with Pork, United States, 2009–2018

Appendix 1

Appendix 1 Table 1. Resistance determinants to first-line antimicrobial drugs or colistin and associated plasmids among sequenced *Salmonella enterica* serotype 4,[5],12:i:- isolates by membership in a multidrug resistant (MDR) clade in a study of infections associated with pork, United States, 2015–2018*

			Isolates in MDR clade†		Isolates outside MDR clade†	
Antimicrobial drug, resistance determinant	Predicted decreased susceptibility, no. (%)‡	Most frequent plasmids (%)§	ARD with predicted decreased susceptibility, no. (%)*	Most frequent plasmids (%)§	ARD and predicted decreased susceptibility, no. (%)*	Most frequent plasmids (%)§
Azithromycin	N = 14		N = 11		N = 3	
mph(A)	9 (64.3)	IncQ1 (88.9), Col (44.4), IncFII (44.4)	6 (54.5)	IncQ1 (100), Col (66.7), IncC (33.3)	3 (100)	IncFIB (100), IncFII (100), IncFIA (66.7)
erm(42)	2 (14.3)	Col (100), IncQ1 (100)	2 (18.2)	Col (100), IncQ1 (100)	0	
mef(B)	2 (14.3)	IncFIB (100), IncFIC (100)	2 (18.2)	IncFIB (100), IncFIC (100)	0	
mph(E)	1 (7.1)	Col (100), IncHI2 (100), IncHI2A (100)	1 (9.1)	Col (100), IncHI2 (100), IncHI2A (100)	0	
msr(E)	1 (7.1)	Col (100), IncHI2 (100), IncHI2A (100)	1 (9.1)	Col (100), IncHI2 (100), IncHI2A (100)	0	
Ceftriaxone	N = 135		N = 113		N = 22	
blaCMY-2	60 (44.4)	IncI1/I (58.3), IncQ1 (50), IncC (35)	43 (38.1)	IncQ1 (69.8), IncI1/I (48.8), IncC (44.2)	17 (77.3)	IncFIB (88.2), IncFII (88.2), IncI1/I (82.4)
blaSHV-12	46 (34.1)	IncHI2 (100), IncHI2A (100), IncQ1 (89.1)	46 (40.7)	IncHI2 (100), IncHI2A (100), IncQ1 (89.1)	0	
blaCTX-M-55	21 (15.6)	IncQ1 (61.9), IncC (52.4), Col (42.9)	21 (18.6)	IncQ1 (61.9), IncC (52.4), Col (42.9)	0	
blaCTX-M-124	3 (2.2)	IncFIA (100), IncFIB (100), IncFII (100)	0		3 (13.6)	IncFIA (100), IncFIB (100), IncFII (100)
blaCTX-M-14	3 (2.2)	IncHI2 (66.7), IncHI2A (66.7), IncQ1 (66.7)	3 (2.7)	IncHI2 (66.7), IncHI2A (66.7), IncQ1 (66.7)	0	

Antimicrobial drug, resistance determinant	Predicted decreased susceptibility, no. (%)‡	Most frequent plasmids (%)§	Isolates in MDR clade†		Isolates outside MDR clade†	
			ARD with predicted decreased susceptibility, no. (%)*	Most frequent plasmids (%)§	ARD and predicted decreased susceptibility, no. (%)*	Most frequent plasmids (%)§
blaCTX-M-65	1 (0.7)	IncFIB (100), IncFII (100), IncHI2 (100)	0		1 (4.5)	IncFIB (100), IncFII (100), IncHI2 (100)
blaTEM-93	1 (0.7)	IncFIA (100), IncFIB (100), IncFII (100)	0		1 (4.5)	IncFIA (100), IncFIB (100), IncFII (100)
Ciprofloxacin	N = 320		N = 271		N = 49	
qnrB19	222 (69.4)	Col (94.6), IncQ1 (70.7), IncFII (16.7)	190 (70.1)	Col (98.9), IncQ1 (75.8), IncX1 (13.2)	32 (65.3)	IncFIB (96.9), IncFII (96.9), Col (68.8)
gyrA	40 (12.5)	#	24 (8.9)	#	16 (32.7)	#
qnrB2	27 (8.4)	IncHI2 (100), IncHI2A (100), IncQ1 (88.9)	27 (10)	IncHI2 (100), IncHI2A (100), IncQ1 (88.9)	0	
qnrS1	27 (8.4)	IncQ1 (66.7), Col (40.7), IncC (40.7)	27 (10)	IncQ1 (66.7), Col (40.7), IncC (40.7)	0	
aac(6')-Ib-cr	24 (7.5)	IncHI2 (95.8), IncHI2A (95.8), IncQ1 (83.3)	24 (8.9)	IncHI2 (95.8), IncHI2A (95.8), IncQ1 (83.3)	0	
qnrA1	2 (0.6)	IncFIB (50), IncFII (50), IncHI2 (50)	1 (0.4)	IncHI2 (100), IncHI2A (100), IncQ1 (100)	1 (2)	IncFIB (100), IncFII (100), IncI1/I (100)
qnrB5	1 (0.3)	Col (100), IncQ1 (100)	1 (0.4)	Col (100), IncQ1 (100)	0	
qnrD1	1 (0.3)	Col (100), IncQ1 (100), IncY (100)	1 (0.4)	Col (100), IncQ1 (100), IncY (100)	0	
qnrVC1	1 (0.3)	Col (100), IncC (100), IncQ1 (100)	1 (0.4)	Col (100), IncC (100), IncQ1 (100)	0	
Colistin	N = 5		N = 3		N = 2	
mcr-1.1	3 (60)	IncI2 (66.7), IncQ1 (66.7), Col (33.3)	2 (66.7)	IncI2 (100), IncQ1 (100), Col (50)	1 (50)	IncFIB (100), IncFII (100), IncI1/I (100)
mcr-3.24	1 (20)	IncC (100)	1 (33.3)	IncC (100)	0	
pmrB	1 (20)	#	0		1 (50)	#

*ARD, antimicrobial resistance determinants; Inc, incompatibility type; MDR, multidrug resistant.
†MDR clade contained isolates with range of 0–108 alleles between isolates by core genome multilocus sequence typing.
‡Percentages for some drugs do not add up to 100% because predicted resistance to a particular agent can have multiple genetic determinants in the same isolate.
§Some isolates might have plasmids with multiple incompatibility types.
#Mutational resistance determinant; plasmids not listed.

Appendix 1 Table 2. Predicted resistance of sequenced isolates of *Salmonella enterica* serotype 4,[5],12:i:- by membership in a multidrug resistant clade associated with ASSuT resistance, United States, 2015–2018*

Drug or resistance pattern	All isolates, n = 3,036	MDR clade, n = 2,087†	Not MDR clade, n = 949†	p value‡
Ampicillin	1,777 (58.5)	1,712 (82)	65 (6.8)	<0.001
Streptomycin	1,771 (58.3)	1,688 (80.9)	83 (8.7)	<0.001
Sulphamethoxazole	1,758 (57.9)	1,685 (80.7)	73 (7.7)	<0.001
Tetracycline	2,085 (68.7)	1,955 (93.7)	130 (13.7)	<0.001
Ciprofloxacin	320 (10.5)	271 (13)	49 (5.2)	<0.001
Ceftriaxone	135 (4.4)	113 (5.4)	22 (2.3)	<0.001
Cefoxitin	60 (2)	43 (2.1)	17 (1.8)	0.622
Ampicillin/clavulanic acid	60 (2)	43 (2.1)	17 (1.8)	0.622
Azithromycin	14 (0.5)	11 (0.5)	3 (0.3)	0.427
Erythromycin	62 (2)	59 (2.8)	3 (0.3)	<0.001
Gentamicin	163 (5.4)	139 (6.7)	24 (2.5)	<0.001
Amikacin	54 (1.8)	54 (2.6)	0 (0)	<0.001
Tobramycin	92 (3)	91 (4.4)	1 (0.1)	<0.001
Chloramphenicol	160 (5.3)	132 (6.3)	28 (3)	<0.001
Rifampin	4 (0.1)	4 (0.2)	0 (0)	0.177
Trimethoprim	174 (5.7)	117 (5.6)	57 (6)	0.660
Colistin	5 (0.2)	3 (0.1)	2 (0.2)	0.673
ASSuT§	1,645 (54.2)	1,612 (77.2)	33 (3.5)	<0.001
Any resistance¶	2,215 (73)	2,045 (98)	170 (17.9)	<0.001
First-line agents#	404 (13.3)	333 (16)	71 (7.5)	<0.001

*Values represent no. (%) except where otherwise indicated. Predicted resistance from identified antibiotic resistance determinants using ResFinder; resistance or decreased susceptibility defined using CLSI criteria or ≥32 µg/mL for azithromycin. MDR, multidrug-resistant.
†MDR clade contained isolates with range of 0–108 alleles between isolates by core genome multilocus sequence typing.
‡p value for isolates in the MDR clade compared with isolates not in the MDR clade, calculated as difference between proportions.
§Defined as resistance to ampicillin, streptomycin, sulphamethoxazole, tetracycline.
¶Defined as any predicted resistance or decreased susceptibility.
#Defined as predicted resistance or decreased susceptibility to ciprofloxacin, ceftriaxone, or azithromycin.

Appendix 1 Table 3. Changes in proportion of nontyphoidal *Salmonella* isolates showing any serotype 4,[5],12:i:-, United States, 2009–2018*

Isolate subgroup	Overall period, 2009–2018	Early period, 2009–2013	Late period, 2014–2018	p value†
ASSuT-positive‡	440/23,175 (1.9)	126/11,139 (1.1)	314/12,036 (2.6)	<0.001
ASSuT-negative‡	639/23,175 (2.8)	350/11,139 (3.1)	289/12,036 (2.4)	<0.001
Linked to MDR clade§	449/23,077 (1.9)	113/11,093 (1)	336/11,984 (2.8)	<0.001
Not linked to MDR clade§	532/23,077 (2.3)	317/11,093 (2.9)	215/11,984 (1.8)	<0.001

*Data represent no. in subgroup/total number (%). Surveillance data from National Antimicrobial Resistance Monitoring System (NARMS, https://www.cdc.gov/narms/index.html). Data assessed by resistance pattern and relatedness to a multidrug resistant clade. ASSuT, resistance to ampicillin, streptomycin, sulphamethoxazole, and tetracycline; MDR, multidrug-resistant.
†p value for isolates collected during 2014–2018, compared with isolates collected during 2009–2013, calculated as difference between proportions
‡ASSuT resistance determined using testing methods reported for the National Antimicrobial Resistance Monitoring System and CLSI criteria.
§Isolates were linked to the MDR clade if identified to have compatible pulse-field gel electrophoresis patterns.

Appendix 1 Table 4. Changes in the proportions of *Salmonella* 4,[5],12:i:- isolates in NARMS surveillance with different resistance patterns—United States, 2009–2018*

Drug or resistance pattern	2009–2018, n = 1,079	2009–2013, n = 476	2014–2018, n = 603	p value†
Ampicillin	487 (45.1)	143 (30)	344 (57)	<0.001
Streptomycin	492 (45.6)	146 (30.7)	346 (57.4)	<0.001
Sulphamethoxazole	492 (45.6)	146 (30.7)	346 (57.4)	<0.001
Tetracycline	546 (50.6)	164 (34.5)	382 (63.3)	<0.001
Ciprofloxacin	66 (6.1)	5 (1.1)	61 (10.1)	<0.001
Ceftriaxone	31 (2.9)	10 (2.1)	21 (3.5)	0.177
Azithromycin	3 (0.3)	2 (0.4)	1 (0.2)	0.431
Gentamicin	38 (3.5)	14 (2.9)	24 (4)	0.358
Chloramphenicol	34 (3.2)	11 (2.3)	23 (3.8)	0.16
Trimethoprim-sulfamethoxazole	27 (2.5)	6 (1.3)	21 (3.5)	0.02
ASSuT‡	440 (40.8)	126 (26.5)	314 (52.1)	<0.001
First-line agents§	91 (8.4)	16 (3.4)	75 (12.4)	<0.001

*Values represent no. (%). Resistance or decreased susceptibility was defined using CLSI criteria or ≥32 µg/mL for azithromycin. Azithromycin testing began in 2011; no results are available for 2009–2010.
†p value for isolates collected during 2014–2018, compared with isolates collected during 2009–2013, calculated as difference between proportions.
‡Resistance to ampicillin, streptomycin, sulfamethoxazole, and tetracycline.
§Defined as resistance or decreased susceptibility to ciprofloxacin, ceftriaxone, or azithromycin.

Appendix 1 Table 5. Characteristics of *Salmonella enterica* 4,[5],12:i:- Infections—FoodNet Sites, 2009–2018 (excluding 222 infections linked to outbreaks)*

Characteristics	4,5,12:i:-, n = 3,772	Other nontyphoidal *Salmonella*, n = 65,518	OR (95% CI)	aOR (95% CI)‡	S. 4,5,12:i:-, PFGE linked to MDR clade†		OR (95% CI)	aOR (95% CI)‡
					Linked, n = 1,427	Not linked, n = 1,733		
Age group, y	N = 3,550	N = 61,084			N = 1,305	N = 1,643		
<1	243 (6.8)	5,972 (9.8)	1	1	78 (6)	117 (7.1)	1	1
1–17	1,370 (38.6)	17,584 (28.8)	1.91 (1.67–2.2)	1.92 (1.65–2.23)	487 (37.3)	637 (38.8)	1.15 (0.84–1.56)	1.16 (0.82–1.64)
18–64	1,509 (42.5)	28,825 (47.2)	1.29 (1.12–1.48)	1.32 (1.14–1.54)	589 (45.1)	684 (41.6)	1.29 (0.95–1.76)	1.63 (1.16–2.3)
>65	428 (12.1)	8,703 (14.2)	1.21 (1.03–1.42)	1.31 (1.1–1.56)	151 (11.6)	205 (12.5)	1.1 (0.77–1.58)	1.36 (0.92–2.02)
Sex	N = 3,549	N = 60,996			N = 1,305	N = 1,643		
M	1,776 (50)	28,798 (47.2)	1	1	644 (49.3)	825 (50.2)	1	1
F	1,773 (50)	32,198 (52.8)	0.89 (0.83–0.96)	0.93 (0.86–1.00)	661 (50.7)	818 (49.8)	1.04 (0.90–1.2)	1.02 (0.87–1.19)
Race, ethnicity	N = 3,078	N = 50,595			N = 1,146	N = 1,416		
White, nonHispanic	1,803 (58.6)	33,512 (66.2)	1	1	585 (51)	946 (66.8)	1	1
Black, nonHispanic	533 (17.3)	7,442 (14.7)	1.33 (1.20–1.47)	1.26 (1.14–1.40)	212 (18.5)	206 (14.5)	1.66 (1.34–2.07)	1.76 (1.41–2.19)
Hispanic	443 (14.4)	6,066 (12)	1.36 (1.22–1.51)	1.26 (1.13–1.40)	204 (17.8)	165 (11.7)	2.00 (1.59–2.52)	2.18 (1.72–2.76)
Other, nonHispanic	299 (9.7)	3,575 (7.1)	1.55 (1.37–1.77)	1.44 (1.27–1.64)	145 (12.7)	99 (7)	2.37 (1.8–3.12)	2.59 (1.95–3.43)
International travel	218/3,065 (7.1)	4,788/47,445 (10.1)	0.68 (0.59–0.79)	0.70 (0.6–0.81)	106/1,159 (9.1)	77/1,415 (5.4)	1.75 (1.29–2.37)	1.68 (1.21–2.33)
Clinical outcome								
Hospitalized	1,010/3,446 (29.3)	17,097/59,209 (28.9)	1.02 (0.95–1.10)	1.06 (0.98–1.15)	365/1,279 (28.5)	471/1,591 (29.6)	0.95 (0.81–1.12)	0.88 (0.73–1.05)
Died	16/3,448 (0.5)	247/59,311 (0.4)	1.11 (0.67–1.85)	1.13 (0.64–1.99)	6/1,270 (0.5)	7/1,596 (0.4)	1.08 (0.36–3.21)	1.29 (0.38–4.35)
Food consumed§								
Beef	405/626 (64.7)	5,602/9,190 (61)	1.17 (0.99–1.39)	1.14 (0.95–1.37)	194/304 (63.8)	151/230 (65.7)	0.92 (0.64–1.32)	0.93 (0.62–1.38)
Pork	277/613 (45.2)	3,307/8,934 (37)	1.40 (1.19–1.65)	1.37 (1.15–1.64)	153/296 (51.7)	87/227 (38.3)	1.72 (1.21–2.45)	1.75 (1.19–2.57)
Chicken	498/683 (72.9)	7,282/9,877 (73.7)	0.96 (0.81–1.14)	0.78 (0.64–0.94)	251/335 (74.9)	187/258 (72.5)	1.13 (0.78–1.64)	1.16 (0.77–1.75)
Turkey	162/640 (25.3)	2,198/9,344 (23.5)	1.10 (0.92–1.33)	1.04 (0.85–1.26)	83/311 (26.7)	54/237 (22.8)	1.23 (0.83–1.83)	1.38 (0.90–2.11)

*aOR, adjusted odds ratio; OR, odds ratio;
†Excluded 602 isolates without pulsed-field gel electrophoresis (PFGE) information. Among sequenced clinical PulseNet isolates collected during 2015–2018 that also had PFGE information, 2,070/3,007 (69%) belonged to a clade associated with ASSuT resistance that contained 275 PFGE patterns; these patterns were present in 7/937 (0.8%) isolates outside the clade.
‡Adjusted for age, sex, race, and ethnicity
§Foods reported consumed in the 7 d before illness onset.

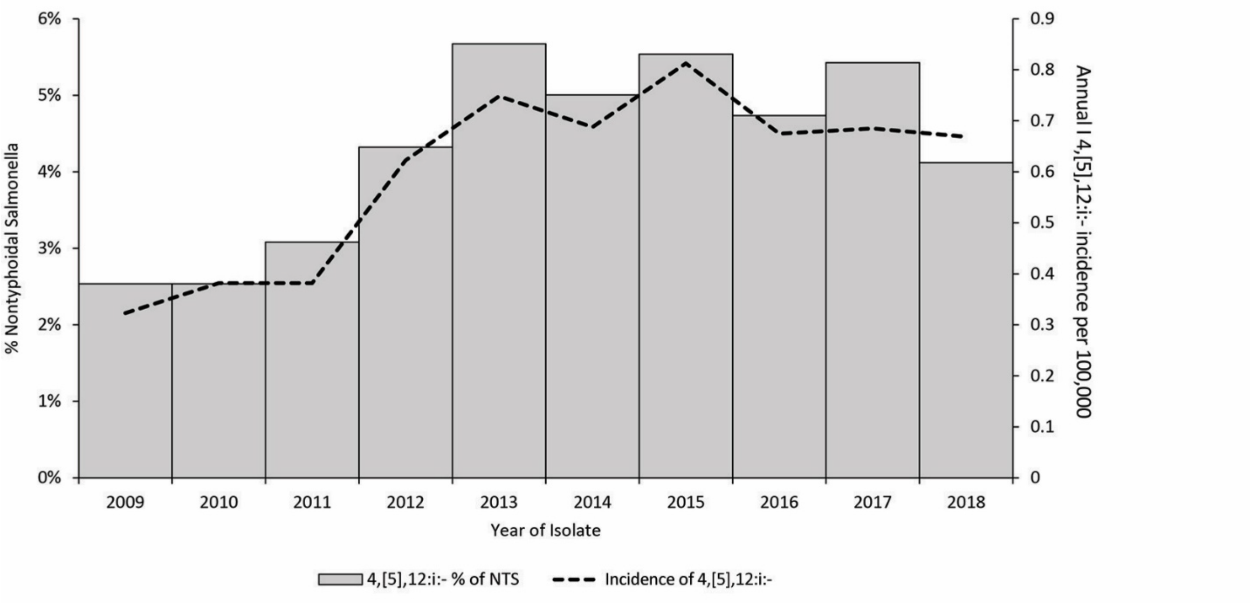

Appendix 1 Figure 1. Incidence of reported multidrug-resistant *Salmonella enterica* serotype 4,[5],12:i:- and other nontyphoidal *Salmonella* in a study of 4,[5],12:i:- infections associated with pork, United States, 2009–2018. NTS, nontyphoidal *Salmonella*.

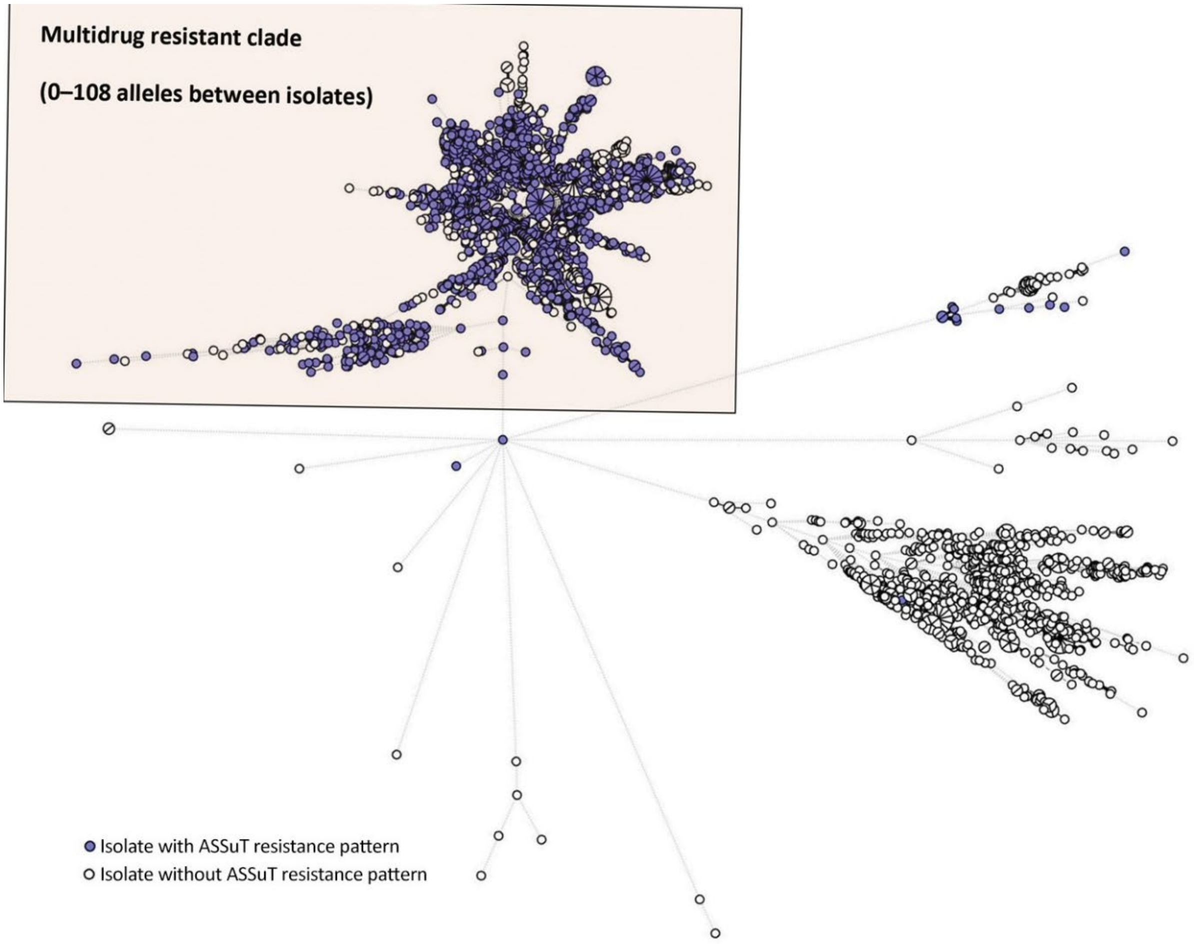

Appendix 1 Figure 2. Phylogenetic clustering and predicted resistance of multidrug-resistant *Salmonella enterica* serotype 4,[5],12:i:-, United States, 2009–2018. Minimal spanning tree constructed by using core genome multilocus sequence typing for sequenced *S. enterica* serotype 4,[5],12:i:- isolates in the PulseNet database. ASSuT isolates are those with resistance genes *bla*TEM-1B, *strA*-*strB*, *sul2*, and *tet*(*B*) that together confer resistance to ampicillin, streptomycin, sulphamethoxazole, and tetracycline. Predicted resistance to antimicrobial drugs was assigned by ResFinder and PointFinder drug keys in the National Antimicrobial Resistance Monitoring System (NARMS; https://github.com/StaPH-B/resistanceDetectionCDC).

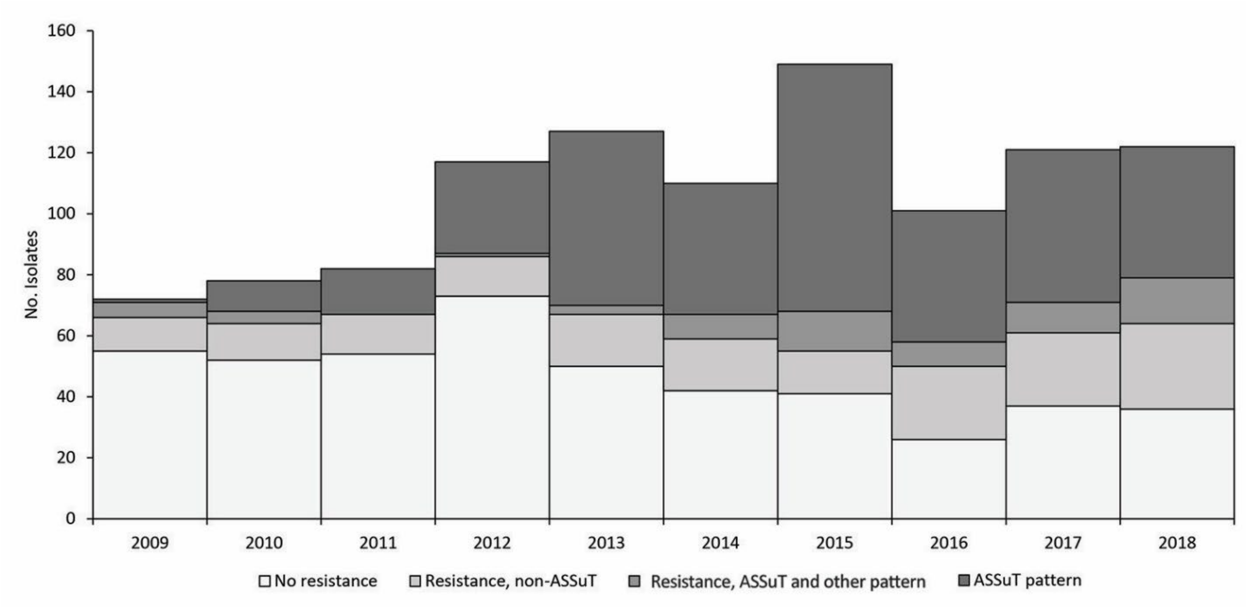

Appendix 1 Figure 3. Number and resistance patterns of multidrug-resistant *Salmonella enterica* serotype 4,[5],12:i:- isolates reported in NARMS per year, United States, 2009–2018. ASSuT, resistance to ampicillin, streptomycin, sulfamethoxazole, and tetracycline by antimicrobial susceptibility testing using standard methods as part of the National Antimicrobial Resistance Monitoring System (NARMS).

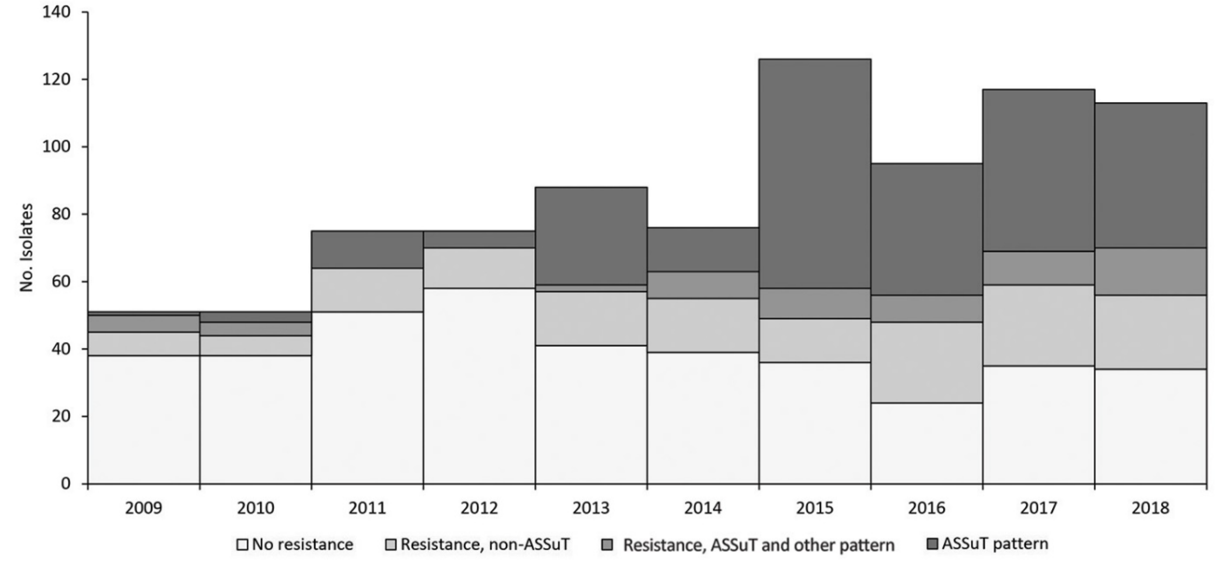

Appendix 1 Figure 4. Number and resistance patterns of nonoutbreak-associated multidrug-resistant *Salmonella enterica* serotype 4,[5],12:i:- isolates reported in NARMS per year United States, 2009–2018. Data excludes 212 isolates linked to outbreaks. ASSuT, resistance to ampicillin, streptomycin, sulfamethoxazole, and tetracycline by antimicrobial susceptibility testing using standard methods as part of the National Antimicrobial Resistance Monitoring System (NARMS).

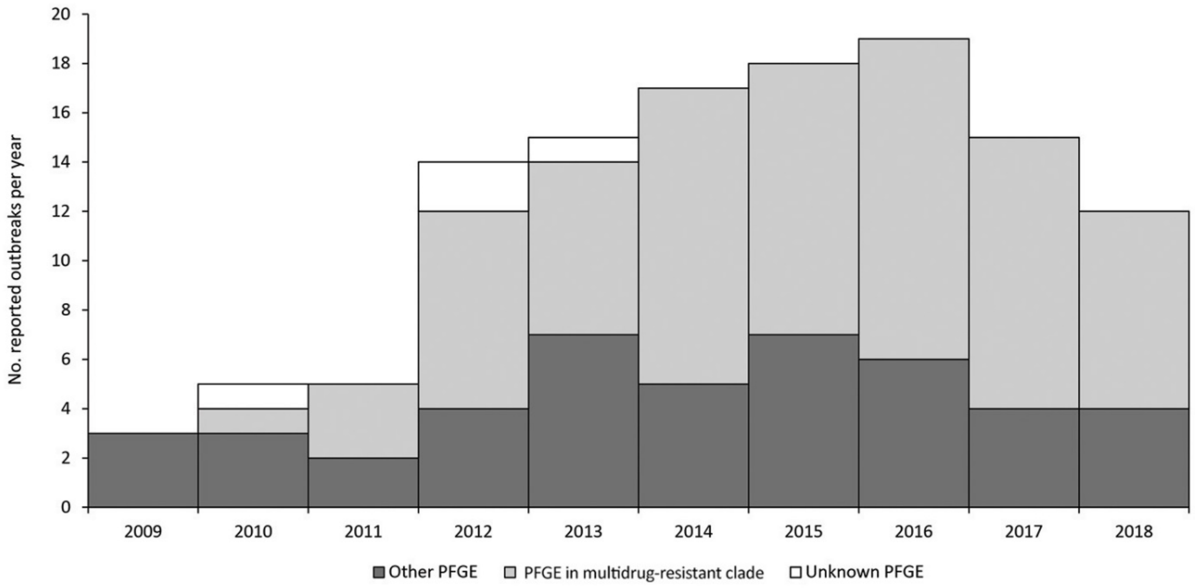

Appendix 1 Figure 5. Number of multidrug-resistant *Salmonella enterica* serotype 4,[5],12:i:- outbreaks reported in NORS per year, United States, 2009–2018. Outbreaks are based on PFGE patterns. ASSuT clade, phylogenetic clade associated with resistance to ampicillin, streptomycin, sulfamethoxazole, and tetracycline; NORS, National Outbreak Reporting System; PFGE, pulse-field gel electrophoresis.
